# Avian Incubation Inhibits Growth and Diversification of Bacterial Assemblages on Eggs

**DOI:** 10.1371/journal.pone.0004522

**Published:** 2009-02-19

**Authors:** Matthew D. Shawkey, Mary K. Firestone, Eoin L. Brodie, Steven R. Beissinger

**Affiliations:** 1 Department of Environmental Science, Policy and Management, Ecosystem Science Division, University of California, Berkeley, California, United States of America; 2 Ecology Department, Earth Sciences Division, Lawrence Berkeley National Laboratory, Berkeley, California, United States of America; University of Alabama, United States of America

## Abstract

Microbial infection is a critical source of mortality for early life stages of oviparous vertebrates, but parental defenses against infection are less well known. Avian incubation has been hypothesized to reduce the risk of trans-shell infection by limiting microbial growth of pathogenic bacteria on eggshells, while enhancing growth of commensal or beneficial bacteria that inhibit or competitively exclude pathogens. We tested this hypothesis by comparing bacterial assemblages on naturally incubated and experimentally unincubated eggs at laying and late incubation using a universal 16S rRNA microarray containing probes for over 8000 bacterial taxa. Before treatment, bacterial assemblages on individual eggs from both treatment groups were dissimilar to one another, as measured by clustering in non-metric dimensional scaling (NMDS) ordination space. After treatment, assemblages of unincubated eggs were similar to one another, but those of incubated eggs were not. Furthermore, assemblages of unincubated eggs were characterized by high abundance of six indicator species while incubated eggs had no indicator species. Bacterial taxon richness remained static on incubated eggs, but increased significantly on unincubated eggs, especially in several families of Gram-negative bacteria. The relative abundance of individual bacterial taxa did not change on incubated eggs, but that of 82 bacterial taxa, including some known to infect the interior of eggs, increased on unincubated eggs. Thus, incubation inhibits all of the relatively few bacteria that grow on eggshells, and does not appear to promote growth of any bacteria.

## Introduction

Microbial infection is a primary source of mortality for early life stages of oviparous vertebrates [1], and this selection pressure has driven the evolution of a suite of morphological and behavioral defenses in parents. Eggs themselves can be viewed as matrices of defense against microbial infection. The tough outer layer provides physical defense and the inner contents, such as albumen in birds [2], [3] and egg jelly in frogs [4], provide chemical defense via antimicrobial proteins. Antimicrobial peptides have been identified in eggs of a wide variety of animal taxa [2], [4], [5].

A complementary parental strategy to reduce the risk of infection is to inhibit growth of pathogenic bacteria on the outer surface of the egg, while enhancing growth of commensal or beneficial bacteria that inhibit or competitively exclude pathogens. Some crustaceans chemically enhance the growth of bacteria that inhibit fungal infection of their eggs [6]. Evidence for comparable manipulations of bacteria has been hypothesized for birds. Antibiotic-producing gram-positive *Enterococcus* spp. occur in the preen gland of hoopoes *Upupa epops* [7] and red-billed woodhoopoes *Phoeniculus purpureus* [8], and it has been suggested that application of oil containing these bacteria to eggs may help defend them against pathogens [7].

Avian incubation can dramatically inhibit total culturable microbial growth on eggshells [9]. These authors found that incubation primarily inhibited gram-negative enterics that can penetrate the shell and infect egg contents, but either promotes or does not inhibit the growth of gram-positive rods that infect eggs less frequently [10], [11], [12], [13], [14], [15]. However, Cook *et al.* [9] did not address the effects of incubation on complete microbial assemblages because they used standard culture-based microbiological methods, which identify less than 1% of environmental microbes [16].

Here we use culture-independent methods to test whether birds selectively inhibit and promote bacterial growth during incubation. We use PhyloChips, high-density oligonucleotide microarrays containing multiple DNA probes for over 8,000 bacterial taxa [17], [18], to compare change over time in the composition and relative abundance of bacteria on naturally incubated and experimentally unincubated eggs. Based on previous work [9], we predicted that relative abundance and diversity of bacteria known to infect eggs such as enterics (family *Enterobacteriaceae*; [10], [11], [12], [13], [14], [15]) would increase on unincubated but not incubated eggs, and predicted the opposite pattern for apparently harmless bacteria like Gram-positive rods and cocci [10], [11], [12], [13], [14], [15].

## Results

Moisture was more common on unincubated than incubated eggshells. All eggs in both groups were dry at laying. At late incubation, however, all unincubated eggs were wet and all incubated eggs were dry (Fisher's exact test, n = 12, *p*<0.01).

We detected 1492 unique taxa in 315 subfamilies, 256 families, 138 orders, 72 classes, and 38 phyla in at least one of the 24 total samples. A complete listing of these taxa is presented in the Supplementary Material (Results S1).

**Figure 1:**
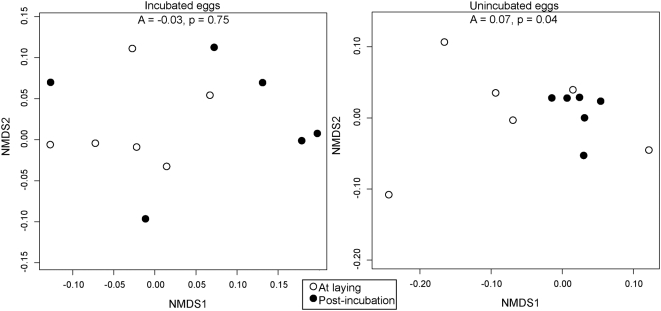
Scatterplots showing placement within non-dimensional metric scaling ordination space of bacterial assemblages on shells of unincubated and incubated eggs at laying and after 12 days. NMDS is a nonparametric ordination technique that maps ranked data non-linearly onto ordination space using both taxa composition and abundance [33]. Here, the assemblage data (composition and relative abundance of taxa) were used to assign a position in ordination space to each sample. Samples with similar assemblages were positioned close to one another in ordination space, while samples with dissimilar assemblages were positioned further apart. To test whether assemblage composition changed over time on incubated or unincubated eggs, we compared positions in ordination space of samples taken before and after treatment in each experimental group. We tested for significant dissimilarity of these positions using a multi-response permutation procedure (MRPP), a nonparametric method for testing group differences that is not constrained by distributional assumptions [34]. The MRPP provides a measure of effect size (A) from 0–1 for within-group homogeneity. Significance of A is tested using a randomization test. *A* and *p* values from multi-response permutation procedures are presented at the top of each panel.

NMDS and MRPP analyses revealed that bacterial assemblages remained constant over time on incubated eggs (Figure 1; A = −0.03, p = 0.75), but changed significantly on unincubated eggs (Figure 1; A = 0.07, p = 0.04). Assemblages on incubated eggs did not change their random arrangement in NMDS ordination space over time, while unincubated eggs were randomly arranged before treatment and became less variable after treatment (Figure 1). This shift towards a uniform bacterial assemblage on unincubated eggs was reflected by the presence of 6 significant Dufrene-Legendre indicator taxa on unincubated eggs after treatment, and none on incubated eggs (Table 1). Thus, unincubated eggs have some characteristic taxon abundances that were not exhibited in incubated eggs. All of these indicator species were significantly more abundant on unincubated than on incubated eggs (Table 1).

**Table 1:** Bacterial taxa with significant Dufrene-Legendre indicator values (IndVal; scale from 0–1) on incubated and unincubated eggs 12 days after egg laying.

Family	Representative taxon in GenBank	Accesssion #	Treatment group	IndVal	*P*	*t*	*P*≤
*Pseudomonadaceae*	*Pseudomonas stutzeri* subgroup Nitrogen-fixing	9295*	Unincubated	0.66	0.021	2.30	0.045
*Enterobacteriaceae*	*Klebsiella planticola* subgroup Br-35	8770*	Unincubated	0.69	0.035	3.36	0.009
*Nitrosomonadaceae*	*Nitrosomonas multiformis* subgroup clone DT-2.3.	7865*	Unincubated	0.74	0.043	6.21	0.001
*Comamonadaceae*	freshwater clone PRD01b009B	AF289169.1	Unincubated	0.69	0.047	3.41	0.007
*Sphingobacteriaceae*	*Pedobacter* sp. An13	AJ551152.1	Unincubated	0.63	0.044	3.45	0.008
*Streptomycetaceae*	*Streptomyces subrutilus* str. DSM 40445	X80825.1	Unincubated	0.75	0.035	3.81	0.006

Significant values indicate that the abundance of that taxa on one group of eggs is sufficiently distinct to serve as an indicator of that group. The right-most *t* and *p* values are from comparions of abundance between incubated and unincubated eggs 12 days after laying.

* Accession number not available, so OTU code for PhyloChip is presented.

**Figure 2:**
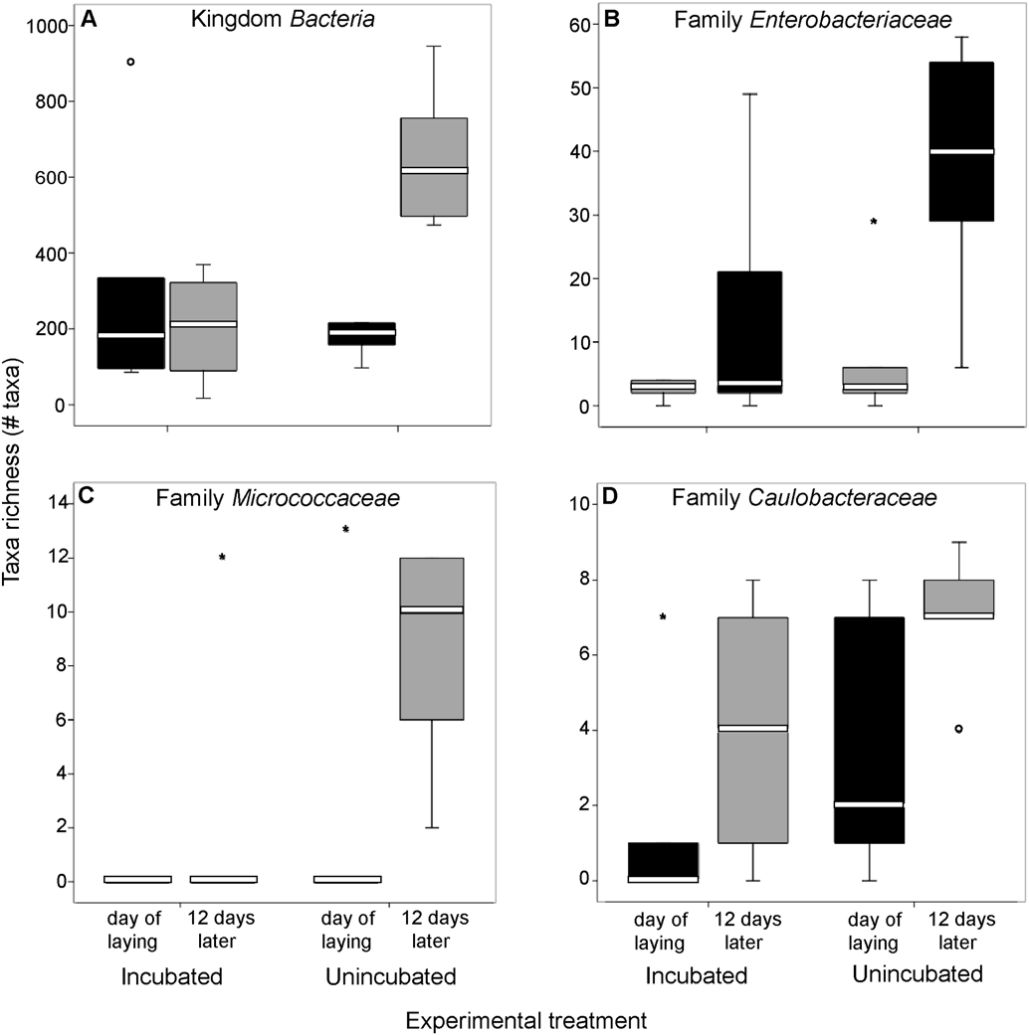
Boxplots of taxa richness of Kingdom *Bacteria* and individual Families within *Bacteria* on incubated or unincubated eggs at laying and after 12 days. Significant (*p*<0.05) differences are indicated with an asterisk. The line within each box represents the median richness, the lower and upper borders of each box are the 25th and 75th percentiles, and the lower and upper bars are the 10th and 90th percentiles. N = 6 in each group.

Temporal changes in taxon richness also differed between incubated and unincubated eggs. Taxon richness of incubated eggs did not significantly differ between early and late incubation at the Kingdom or Family levels (paired t-test: all *p*>0.10; table 2; figure 2a–d). However, taxon richness of unincubated eggs was significantly higher at late incubation than at laying for Kingdom *Bacteria* (paired t-test: *t* = −2.76, *p* = 0.042; table 2; figure 2a) and for Families *Enterobacteriaceae* (*t* = −4.17, *p* = 0.009; table 2; figure 2b), *Micrococcaceae* (*t* = −3.08, *p* = 0.031, table 2; figure 2c), *Frankiaceae* (*t* = −2.71, *p* = 0.041; table 1), *Xanthobacteraceae* (t = −2.71, *p* = 0.041; table 1), and *Caulobacteraceae* (*t* = −2.60, *p* = 0.047; table 2; figure 2d). Taxon richness of the remaining 251 families did not differ significantly between laying and late incubation (all *p*>0.11).

**Table 2:** Comparison of bacterial taxon richness on eggs that were either normally incubated or left unincubated for 12 days.

Taxonomic unit	Incubated eggs before manipulation (±1 S.E.)	Incubated eggs after manipulation (±1 S.E.)	*t*	*p*	Unincubated eggs before manipulation (±1 S.E.)	Unincubated eggs after manipulation (±1 S.E.)	*t*	*p*
	**Taxa richness (# taxa)**				**Taxa richness (# taxa)**			
Kingdom *Bacteria*	298.2±126.5	203.3±56.4	0.65	0.54	261.2±91.8	650.8±76.8	−2.76	**0.04**
Family *Enterobacteriaceae*	2.7±0.7	13.2±7.8	−1.33	0.24	7.2±4.4	37.8±7.7	−4.17	**<0.01**
Family *Micrococcaceae*	0.0±0.0	2.0±2.0	−1.00	0.36	2.2±2.2	8.7±1.6	−3.08	**0.03**
Family *Caulobacteraceae*	1.3±1.5	4.0±1.5	−1.22	0.28	3.3±1.4	7.0±0.7	−2.60	**0.05**
Family *Frankiaceae*	0.0±0.0	0.3±0.2	−1.58	0.18	0.2±0.2	1.0±0.3	−2.71	**0.04**
Family *Xanthobacteraceae*	0.3±0.2	0.3±0.3	0.00	1.00	0.0±0.0	0.8±0.3	−2.71	**0.04**

The *t* and *p* values are from paired t-tests comparing the same eggs immediately after laying and after 12 days. Significant effects (p<0.05) are listed in bold. For brevity, only results from families that significantly or nearly significantly changed in either treatment group are presented. Full results are presented in the Results S1.

**Figure 3:**
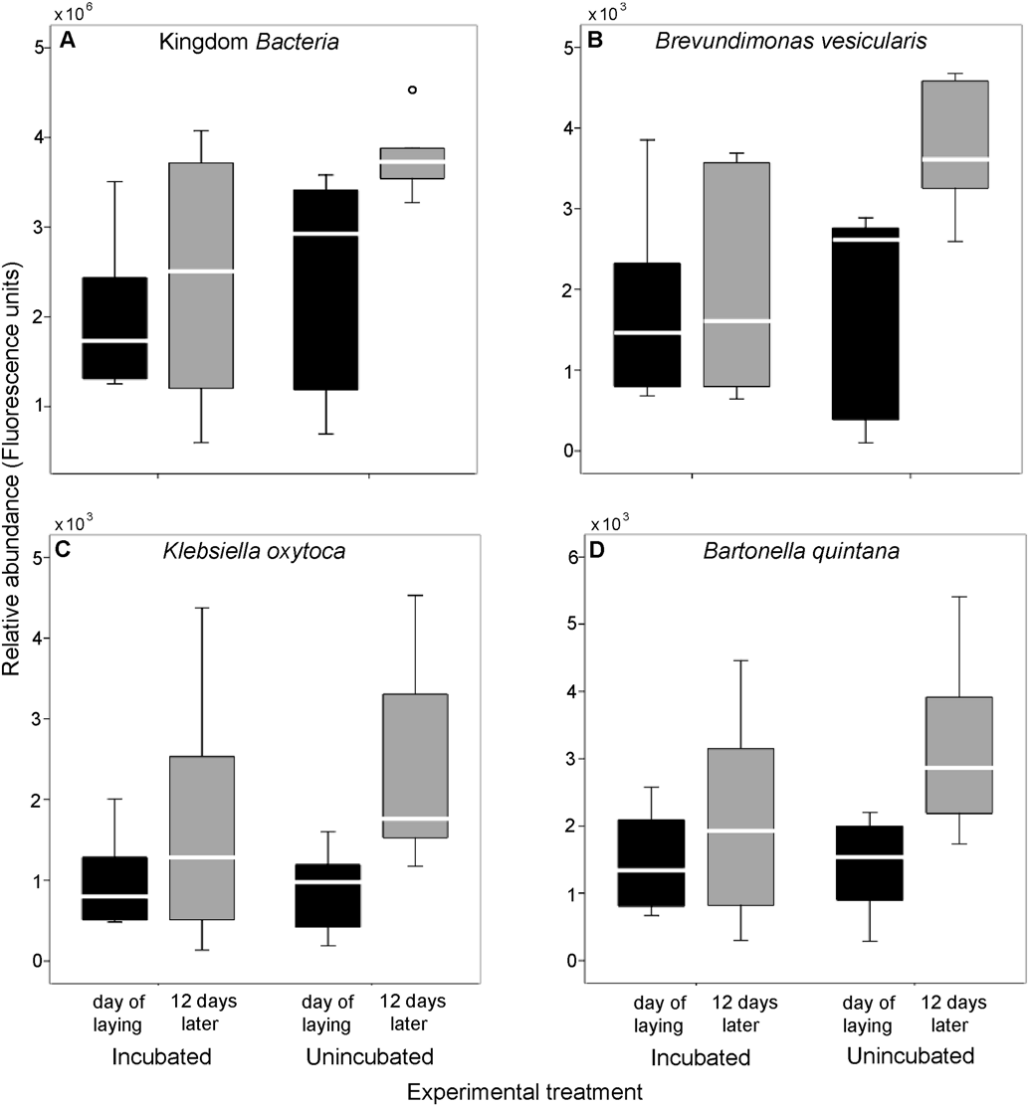
Boxplots of abundance of Kingdom *Bacteria* (measured as DNA concentration) or individual taxa within *Bacteria* (measured as hybridization fluorescence intensity) on incubated or unincubated eggs at laying and after 12 days. Significant (p<0.05) differences are indicated with asterix. The line within each box represents the median richness, and the lower and upper borders of each box are the 25th and 75th percentiles are the lower and upper bars are the 10th and 90th percentiles. N = 6 in each case.

Temporal changes in bacterial abundance did not differ between incubated and unincubated eggs at the Kingdom level, but differed at the taxon level. Total bacterial abundance, measured as DNA concentration, did not significantly differ between laying and late incubation for incubated eggs (paired t-test: *t* = −0.98, *p* = 0.37; figure 3a), or for unincubated eggs (*t* = −1.36, *p* = 0.23; figure 3a). However, relative abundance of some individual taxa increased over time on unincubated, but not incubated, eggs. We analyzed change in relative abundance (PhyloChip fluorescence intensity) of 350 individual taxa. In the incubated group, abundance did not differ significantly between laying and late incubation for any taxa (all *p*>0.10; see figure 3a–d; full data are presented in Results S1, S2). However, in the unincubated group, relative abundance was significantly higher at late incubation than at laying for 81 bacterial taxa (Results S1, S2, figure 3a–d) but did not differ for the remaining 269 taxa (all *p*>0.09). The largest proportions of significant taxa were in the Families *Enterobacteriaceae* (23.2%), *Comamonadaceae* (11.0%), *Caulobacteraceae* (8.5%) and *Sphingomonadaceae* (8.5%).

Separate binomial tests indicated that abundance was not likely to change on incubated eggs (n = 350, p = 0.31) but was likely to increase on unincubated eggs (n = 350, p = 0.035).

## Discussion

This is the first study to use culture-independent techniques to test hypotheses about selective effects of incubation on bacterial assemblages of eggs. Our data demonstrate that avian incubation prevents changes in bacterial assemblages. Assemblages of unincubated eggs diversified and came to resemble one another, while assemblages of incubated eggs remained taxonomically stagnant and dissimilar. Furthermore, relative abundance of nearly one-quarter of examined bacterial taxa increased on unincubated, but not incubated, eggs. This lack of change in assemblage composition and abundance on incubated eggs suggests that incubation is uniformly bacteriostatic and does not promote the growth of any specific bacteria.

We hypothesized that incubation would specifically target harmful bacteria, particularly gram-negative enterics known to infect egg contents. Indeed, species in Family *Enterobacteriaceae* are frequently isolated from the contents of addled eggs [10], [11], [12], [13], [14], [15], and this Family diversified over time on the shells of unincubated eggs. Furthermore, several taxa that grew on unincubated eggs are known opportunistic pathogens in humans or other animals. For example, *Brevundimonas diminuta* (Family *Caulobacteraceae*) and *Klebsiella oxytoca* (Family *Enterobacteriaceae*) are common causes of infection in immunocompromised humans [19], [20]. *Bartonella* spp. (Family *Bartonellaceae*) are the causative agents of trench and cat scratch fevers in humans [21], [22], and are agents of bacteremia in deer [21]. However, many bacteria that are most likely harmless, such as plant-symbiotic *Rhizobiaceae* species, also grew on unincubated eggs. Thus, incubation appeared to inhibit not only harmful bacteria but also all other bacteria that grew on unincubated eggs.

Three results from our study did not support the hypothesis that birds actively apply or promote the growth of harmless bacteria, such as gram-positive rods (e.g. *Bacillus* spp.,[10]) or antibiotic-producing *Enterococcus* spp. [7], [8], to outcompete or inhibit pathogenic bacteria. First, incubated eggs had no significant indicator taxa and did not move towards a similar assemblage composition. Second, no taxon or family significantly increased in abundance or diversity over time on incubated eggs. Third, no taxon significantly decreased on unincubated eggs. Our small sample size prevents us from concluding that incubation has no promotional effects. If such effects exist, however, they are weaker than inhibitory effects. Below we discuss possible mechanisms for this inhibition.

Incubating birds may influence bacterial assemblages by applying oils found on feathers to eggs. Feathers are coated with preen oil that inhibits some bacterial growth [23], [24]. Such oils could both directly control bacterial growth with antimicrobials and indirectly control bacteria by repelling water. Surprisingly, little is known, however, about preen oil's effectiveness either as an antimicrobial or water repellent.

Raising the temperature of eggs through incubation may also remove water from eggs through evaporation [2], [3], [9], [12], [13]. Unincubated eggs were consistently wet and incubated eggs were consistently dry at the end of the 12-day experiment, and this reduction in moisture could explain our results in two ways. First, most bacteria grow better in wet than in dry conditions, and thus overall bacterial abundance should be greater on unincubated eggs. However, our finding that overall abundance does not change with time on either group of eggs does not support this idea. Second, a wet egg surface should lead to an interconnected microbial assemblage, creating consistent interactions between bacteria and thus high homogeneity. By contrast, a dry egg surface should lead to a patchy and spatially separated microbial assemblage with greater variation. This explanation fits the patterns of assemblage change shown in Figure 1 well. Perhaps by preventing homogenization and minimizing bacterial interactions over the egg surface, birds can more effectively prevent infection. This hypothesis could be tested by experimentally adjusting moisture patterns on eggs and measuring relative changes in their bacterial assemblages.

Many other mechanisms, including turning of eggs by parents, the physical abrasion or loss of oxygen potentially caused by incubation could also explain our results. Identifying the relative importance of these and others will be fertile ground for future research. The role of heat is particularly interesting, given that many bacteria grow well at typical incubation temperatures (∼34–37° C). Do other mechanisms compensate for or work in concert with incubation temperatures to reduce microbial load?

We have shown here that incubation in thrashers inhibits growth and diversification of the relatively small number of bacteria that grow well on eggshells. This broad bacteriostatic effect contrasts with the more narrow bactericidal action of antimicrobial peptides applied to eggs by fish [25], [26]. Clarifying the mechanisms by which birds slow the growth of bacteria on eggshells and reduce the risk of microbial infection to developing embryos is a critical area for future work as we move towards understanding the significance of microbial infection to vertebrate evolution.

## Materials and Methods

We performed our experiment in March 2007 on eggs from an established box-nesting population of pearly-eyed thrashers *Magarops fuscatus* in the El Yunque rainforest (18°38′N 65°87′W). See Arendt (1993) and Cook *et al.* (2003) for details of the study site. The pearly-eyed thrasher is an omnivorous, cavity-nesting passerine found in forests throughout Puerto Rico [27]. It typically lays one egg a day until reaching a clutch size of 3 or 4 eggs [28]. Full incubation begins after laying of the penultimate egg, although bouts of incubation may occur earlier (M.I. Cook, unpubl. data), and continues for 14–16 days [29]. Previous studies have shown that microbial infection of eggs can occur in this species (Cook *et al.* 2005b) and that incubation reduces microbial loads on their shells (Cook *et al.* 2005a). All animals were treated in accordance with the guidelines of the Institutional Animal Care and Use Committee of the University of California at Berkeley and with the laws of the USA.

We checked nest boxes between 0900 and 1300 every three days until nests were fully lined (indicating that the bird was about to lay), at which point we checked them daily. The first-laid egg in a nest was randomly designated as either incubated or unincubated, and was used as our sampling unit. To prevent incubation in unincubated nests, we stopped females from using the nest site by securing wire mesh screens over the entrance. We did not manipulate incubated nests, which continued to receive natural incubation by females.

We sampled bacteria on eggshells on the day of laying (hereafter “laying”) and 12 days after laying (hereafter “late incubation”). We chose the laying sample date to avoid effects of early incubation and the late incubation sample date to avoid early hatching of the incubated eggs. Because we predicted a strong effect based on previous work (Cook *et al.* 2005) and because it was expensive to apply PhyloChip methods, our final sample size totaled 24 samples for phylochip analysis from 6 incubated and 6 unincubated eggs. We noted whether eggs were wet or dry (based on visual inspection) at each sampling period.

Wearing sterile gloves, we sampled bacteria on eggshells by removing the egg from the nest and rubbing a sterile swab (FisherBrand, Santa Clara, CA) dipped in sterile phosphate buffered saline (PBS)/0.05% Tween-80 over approximately half of its surface. We used a Sharpie to mark the sampled side of the egg during the pre-incubation visit and sampled the other side during the late incubation visit. These swabs were placed in sterile tubes containing 500 µl of sterile PBS/0.05% Tween-80, sealed, and transported on ice to a lab within 3–4 hours of collection. In the lab, we vortexed each tube three times for ∼5 seconds to remove bacteria from the swab, centrifuged it for 10 minute at 13,000×*g* and then removed the swab. After a second centrifugation for 10 minute at 13,000×*g*, we extracted DNA using the DNeasy® Tissue Kit (Qiagen, Valencia, CA) according to the manufacturer's instructions for Gram-positive bacteria, measured its concentration using a NanoDrop ND-1000 spectrophotometer (Thermo Fisher, Delaware, USA) and used it as a template for PCR amplification of the bacterial 16S rRNA gene.

We used “universal” primers 27F (5′- AGRGTTTGATCMTGGCTCAG - 3′) and 1492R (5′- GGTTACCTTGTTACGACTT - 3′). All PCRs were performed in 50 µl reaction volumes containing (as final concentrations) 1× *ExTaq* buffer (Takara Bio Inc., Japan), 0.8 mM dNTP's, 1.0 mM of each forward and reverse primer, 0.4 mg/ml BSA and 0.02 U/ml *ExTaq* DNA polymerase. All reactions were incubated on a MyCycler thermal cycler (Bio-Rad, Carlsbad, CA) for an initial denaturation step at 95° C for three minutes, followed by 35 cycles of denaturation (95° C, 30 s), annealing (53° C, 30 s) and extension (72° C, 60 s), followed by a final extension step (72° C, 10 min). Three reactions were performed and pooled for each sample. PCR products were purified and concentrated by isopropanol precipitation and concentration was determined by gel electrophoresis comparison to known mass ladders using 2% e-gels (Invitrogen, Carlsbad, CA).

Traditional culture-independent techniques such as clone-library analysis of biomarker genes [16], [30] are frequently time- and cost-intensive, and in even the best circumstances may provide incomplete coverage [31], [32]. The PhyloChip offers a rapid and robust method for determining the presence and relative abundance of known bacterial taxa in samples. Thus, it is ideal for tracking changes in assemblages over time and/or with experimental treatment. Detailed descriptions of PhyloChip construction, validation and methodologies can be found in Brodie *et al.* [17], [18]. Briefly, 500 ng of pooled PCR product was DNaseI fragmented and biotin labeled, and an aliquot was hybridized to custom-made Affymetrix GeneChips (16S PhyloChip). Hybridization was performed at 60 rpm at 48°C overnight. PhyloChip washing and staining were performed according to standard Affymetrix protocols. Each PhyloChip was scanned and recorded as a pixel image, and initial data acquisition and intensity determination were performed using standard Affymetrix software (GeneChip microarray analysis suite, version 5.1). The intensity was recorded as the trimmed average of probe sets as described previously [17]. The positive fraction (PosFrac) was calculated for each probe set as the number of positive probe pairs divided by the total number of probe pairs in a probe set. A taxon was considered present in the sample when over 90% of its assigned probe pairs were positive (PosFrac>0.90).

We used DNA concentration as an index of overall bacterial abundance [18], and the number of taxa present in Kingdom *Bacteria* and in each Family as estimators of richness. Following Brodie *et al.* [17], we used hybridization fluorescence values as indicators of relative abundance of individual taxa. For analyses at the taxon level, we only used the 350 taxa present in at least half of the samples in either treatment. Samples not classified at the Family level were not included.

Our analyses incorporated both multivariate and univariate techniques. First, we used non-metric dimensional scaling [33] to compare similarity in distribution of bacterial taxa on incubated and unincubated eggs before and after treatment. NMDS is a nonparametric ordination technique that maps ranked data non-linearly onto ordination space using both taxa composition and abundance, and can robustly find the underlying gradient of most sets of species responses [33]. Here, the assemblage data (composition and relative abundance of taxa) were used to assign a position in ordination space to each sample. Samples with similar assemblages were positioned close to one another in ordination space, while samples with dissimilar assemblages were positioned further apart. To test whether assemblage composition changed over time on incubated or unincubated eggs, we compared positions in ordination space of samples taken before and after treatment in each experimental group. We tested for significant dissimilarity of these positions using a multi-response permutation procedure (MRPP), a nonparametric method for testing group differences that is not constrained by distributional assumptions [34]. The MRPP provides a measure of effect size (A) from 0–1 for within-group homogeneity. Significance of A is tested using a randomization test. We used the *vegan* package [35] in the R statistical software [36] to carry out these analyses. We used function *metaMDS* for NMDS and function *mrpp* for MRPP. In all cases we used Bray-Curtis dissimilarity as the distance measure.

Second, we used Dufrene-Legendre indicator species analysis [37] to identify bacterial taxa strongly associated with either unincubated or incubated eggs after treatment. These tests give a value from 0–1, indicating the strength of a taxon's association with a particular group, and a significance value generated by a randomization procedure. We used function *duleg* in the *labdsv* [38] package in the R statistical software to carry out these analyses.

Finally, in separate analyses for incubated and unincubated eggs, we dissected the changes observed at the assemblage level among taxa using univariate analyses. We compared richness and relative abundance values between laying and late incubation measurements on the same eggs using paired t-tests. This paired approach is more powerful than comparisons between incubated and unincubated eggs [39]. These analyses were carried out using SPSS version 13. Bonferroni correction to lower false discovery rate is frequently applied when multiple comparisons are made on the same set of data, but such a correction may be overly conservative [40], [41]. If a Bonferroni correction were used here, α would be 0.00014. For less conservative sequential Bonferroni and Dunn-Sidak corrections, α for comparisons would range between 0.05 and 0.00014, with over 85% of comparisons set at α<0.001. We do not use any of these corrections for two reasons. First, the strength of the Phylochip (precise data for >8,500 individual taxa) makes it a statistical challenge that can be dealt with on some level by using the multivariate techniques we employed. However, eliminating univariate comparisons or essentially rendering them meaningless by stipulation of extremely low *p* values would also eliminate the Phylochip's strength. Second, we detected no significant (α = 0.05) differences in either richness or abundance for our control group but many significant differences for our experimental group. All differences were in the same direction, and we have little reason to suspect that they were caused by chance. We therefore argue that such corrections are not needed in this case. Finally, to determine if abundance was more likely to increase or decrease in either group, we used separate binomial tests on incubated and unincubated eggs with direction of change (increase or decrease) as the test variable.

## Supporting Information

Results S1 A spreadsheet listing all of the bacterial taxa recorded as present (PosFrac≥0.9 on the PhyloChip microarray) on at least five of the 24 eggs sampled in this study. Phylum, Class, Order, Family and Subfamily, as well as the OTU number in GreenGenes (http://greengenes.lbl.gov) and accession number GenBank of each taxon are listed. The mean relative abundance ±1 S.E. of each taxon (measured as fluorescence intensity) before and after treatment is listed for both incubated (columns J and K) and unincubated (rows N and O) eggs. Values of t and p for paired t tests are presented for incubated (columns L and M) and unincubated (Columns P and Q) eggs.
(0.17 MB XLS)

Results S2 Complete normalized microarray data file for all the Phylochips used in this study. Key to chip identity is included at the top of the chart.
(2.23 MB XLS)
